# Quantifying fat replacement of muscle by quantitative MRI in muscular dystrophy

**DOI:** 10.1007/s00415-017-8547-3

**Published:** 2017-07-01

**Authors:** Jedrzej Burakiewicz, Christopher D. J. Sinclair, Dirk Fischer, Glenn A. Walter, Hermien E. Kan, Kieren G. Hollingsworth

**Affiliations:** 1 Department of Radiology, C. J. Gorter Center for High Field MRI, Leiden University Medical Centre, Leiden, The Netherlands; 2 MRC Centre for Neuromuscular Diseases, UCL Institute of Neurology, London, UK; 3 Neuroradiological Academic Unit, UCL Institute of Neurology, London, UK; 4 Division of Neuropaediatrics, University of Basel Children’s Hospital, Spitalstrasse 33, Postfach, Basel, 4031 Switzerland; 5 Department of Neurology, University of Basel Hospital, Petersgraben 4, Basel, 4031 Switzerland; 6 Department of Physiology and Functional Genomics, University of Florida, Gainesville, FL 32610 USA; 7 Newcastle Magnetic Resonance Centre, Institute of Cellular Medicine, Newcastle University, Newcastle-upon-Tyne, UK

**Keywords:** Muscular dystrophy, MRI, Quantitative, Duchenne, Muscle, Clinical trial

## Abstract

The muscular dystrophies are rare orphan diseases, characterized by progressive muscle weakness: the most common and well known is Duchenne muscular dystrophy which affects young boys and progresses quickly during childhood. However, over 70 distinct variants have been identified to date, with different rates of progression, implications for morbidity, mortality, and quality of life. There are presently no curative therapies for these diseases, but a range of potential therapies are presently reaching the stage of multi-centre, multi-national first-in-man clinical trials. There is a need for sensitive, objective end-points to assess the efficacy of the proposed therapies. Present clinical measurements are often too dependent on patient effort or motivation, and lack sensitivity to small changes, or are invasive. Quantitative MRI to measure the fat replacement of skeletal muscle by either chemical shift imaging methods (Dixon or IDEAL) or spectroscopy has been demonstrated to provide such a sensitive, objective end-point in a number of studies. This review considers the importance of the outcome measures, discusses the considerations required to make robust measurements and appropriate quality assurance measures, and draws together the existing literature for cross-sectional and longitudinal cohort studies using these methods in muscular dystrophy.

## Introduction: a motivation for quantifying muscle replacement by fat

All muscular dystrophies (MD) are rare orphan diseases, characterized by progressive muscle weakness resulting in functional disability. While the causes for muscle weakness vary between different MDs, they share common histological features, including fibrosis, muscle edema, and fat replacement of muscle tissue. More than 70 distinct variants have been identified, some becoming apparent in early childhood with rapid progression while others are not detected until adulthood with a much slower timescale of progression. Among the muscular dystrophies, Duchenne muscular dystrophy (DMD) is the most common form affecting 1 in 3500–6000 male births [1, 2], tending to show first symptoms at 3–5 years of age and rapid progression leading to loss of ambulation between 10 and 15 years. Deterioration of respiratory function and cardiomyopathy occur in DMD and in many other MDs.

Planning clinical trials of therapy in MDs is challenging for three reasons. First, the progression of the disease does not always follow a linear course when physical abilities are measured. Longitudinal studies using clinical measurements in DMD show an improvement of gross and fine motor function up to the age of 7, a plateau phase up to the age of 10 followed by a phase of rapid decline during which free ambulation is lost [3, 4]. Second, recruitment in rare disease cohorts for large clinical trials is difficult. Third, clinical assessment is dependent on cooperation (especially in children) and the measurements required differ between ambulant and non-ambulant patients.

Therefore, it is essential to have outcome measures that are less dependent on patient cooperation and motivation while being sufficiently sensitive to measure treatment effects even in small and highly heterogeneous patient populations. Quantitative muscle MRI (qMRI) can provide such an outcome measure, as it is non-invasive and can generate a wide range of imaging contrasts over large volumes of muscle. As such, it can visualize and quantify the major hallmarks of muscle degeneration in MDs: muscle hypertrophy and atrophy, muscle edema and inflammation, and the replacement of muscle tissue by fat tissue. Histologically, the content of the muscle fibres is progressively replaced by lipid, resulting in a fatty tissue. On MRI, the overall outline, shape, structure, and appearance of the muscle groups are often preserved despite the extensive fatty tissue content. In contrast to functional scores obtained by physical assessment, qMRI is less biased by patients or observers. Excellent reproducibility of qMRI has been demonstrated in healthy volunteers as well as in patients [5, 6]. In particular, qMRI of fat replacement has the potential to be very valuable as an outcome measure in clinical trials as it has been shown to be more sensitive than clinical evaluation in detecting disease progression [7–12] and effect sizes of fat replacement measured by qMRI are much greater than those of commonly used clinical scores [10, 13]. Thus, when using qMRI of fat replacement as an end-point to demonstrate the effectiveness of a putative novel treatment, the number of patients required is markedly reduced [9, 10]. Although invasive muscle biopsy is sometimes undertaken (often to determine membrane protein expression) and fat content could be estimated from it, it is an unreliable method of measuring fat content due to the small sample volume, the heterogeneous nature of the fat replacement across muscle groups, and the limitations on repeating biopsy in longitudinal studies.

In this review, we review the MRI sequences used to quantify fat replacement of skeletal muscle by imaging and detail the important factors that need to be taken into account to produce accurate results (“Quantitative fat fraction imaging measurements: how is it done?”), compare these with magnetic resonance spectroscopy methods (“T2-corrected spectroscopy to measure muscle fat content”), and provide a survey of longitudinal and cross-sectional studies of muscular dystrophy which have employed variants of these methods (“Studies which have used quantitative fat fraction measurements in muscular dystrophy”). We then consider the quality assurance required for using these methods (“Quality assurance”) and practical considerations for patient positioning, slice prescription, and data analysis (“Patient placement, acquisition time and scan acceleration, practical issues and segmentation”). Most MR vendors have developed products for measuring liver steatosis and we discuss the necessary considerations for using these in skeletal muscle (“Vendor implementations of quantitative protocols to measure PDFF”).

## Quantitative fat fraction imaging measurements: how is it done?

### T1-weighted imaging as fat quantification technique

**Fig. 1:**
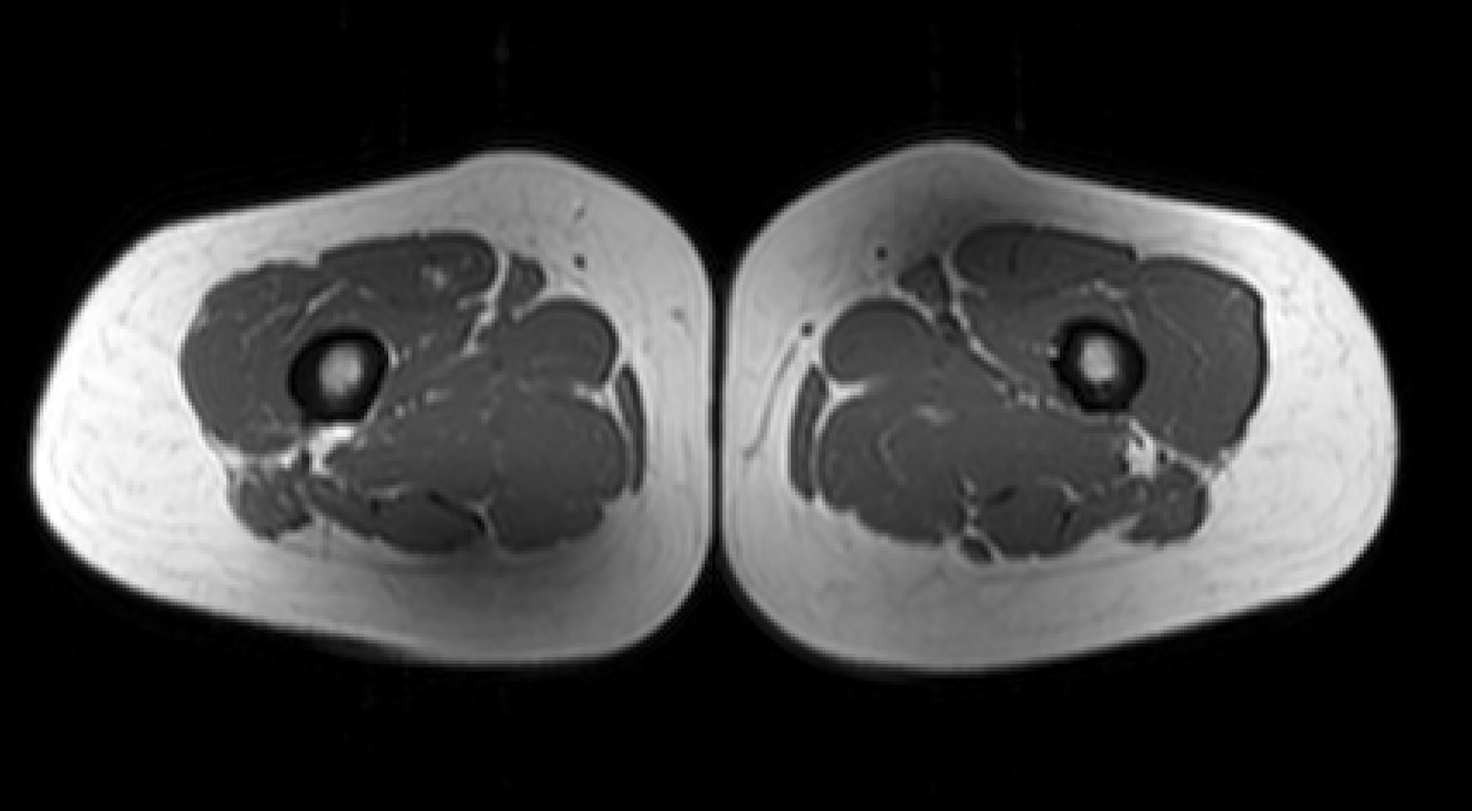
T1-weighted image of dystrophic thigh muscle. The widely varying signal intensity in the uniform subcutaneous fat demonstrates the B_1_ inhomogeneity across the leg at 3.0 T which inhibits the ability of T1-weighted images to monitor disease progression

While T1-weighted MRI is commonly used in routine diagnostic procedures to assess muscle involvement, T1-weighted imaging has several disadvantages when it is used to measure fat replacement. First, the signal intensity does not directly quantify changes in muscle fat content and needs to be referenced, e.g., to the subjects’ bone marrow intensity of the same image section and expressed as a percentage of the bone marrow signal intensity [14]. Second, quantifying muscle signal intensity with T1-weighted imaging is subject to B_1_ and B_0_ inhomogeneities [14], as shown in Fig. 1. Finally, T1-weighted imaging is less sensitive than quantitative MRI at detecting fat changes in muscle of patients with MD [7, 11–13], as described in the next section. Overall, we do not recommend using T1-weighted imaging as a quantitative measure. In the following sections, we instead describe the principles of quantitatively measuring fat using chemical shift imaging techniques.

### Principles of fat fraction measurement using chemical shift differences

**Fig. 2:**
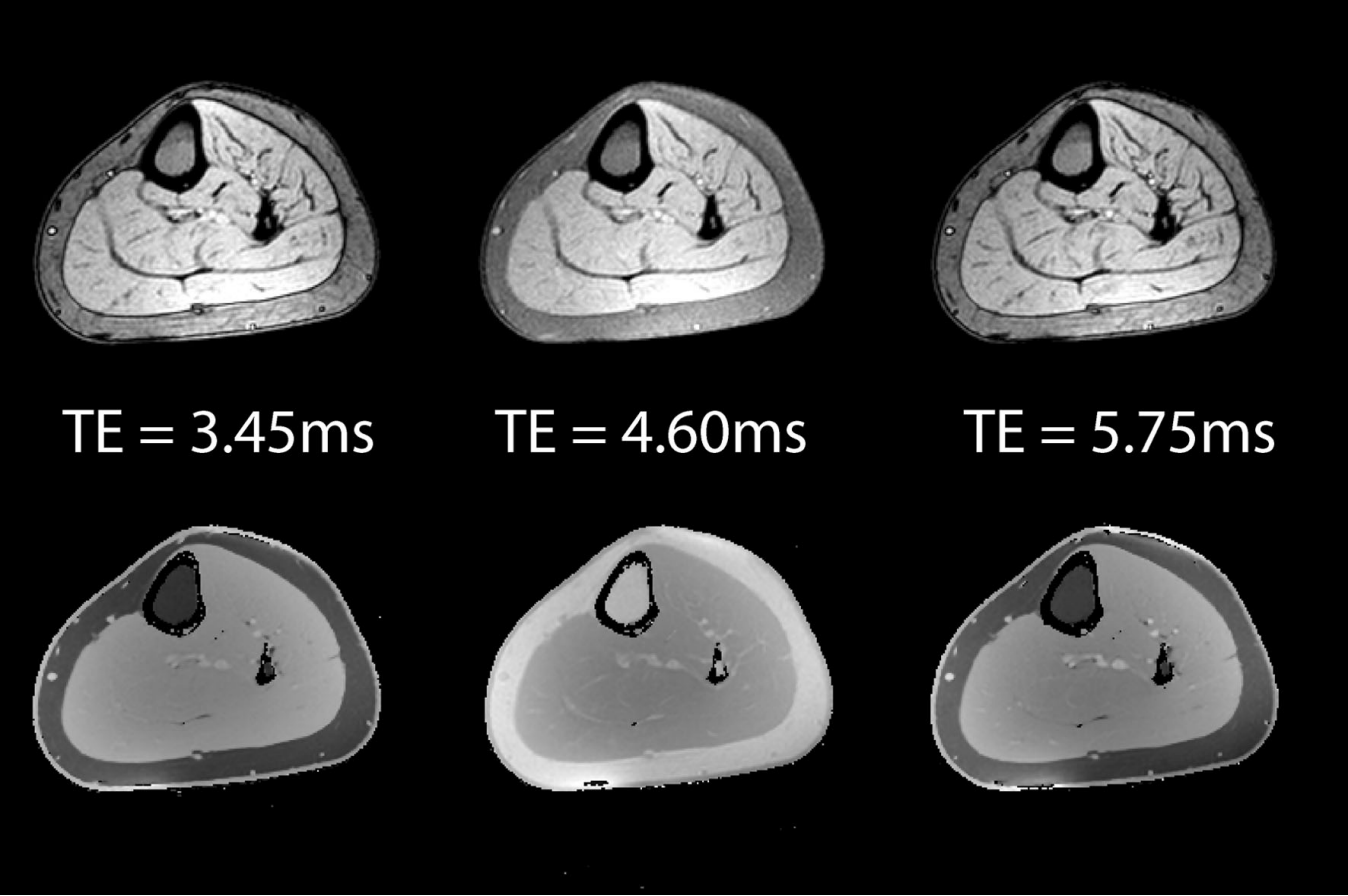
Cross-section through healthy lower leg muscle with a gradient echo sequence using (*left*) out of phase (TE = 3.45 ms), (*middle*) in phase (TE = 4.6 ms), and (*right*) out of phase (TE = 5.75 ms) echo times. The *top row* shows the magnitude signal, while the *bottom shows* the phase. Note the cancellation of the magnitude signal at water–fat boundaries in the out of phase images

**Fig. 3:**
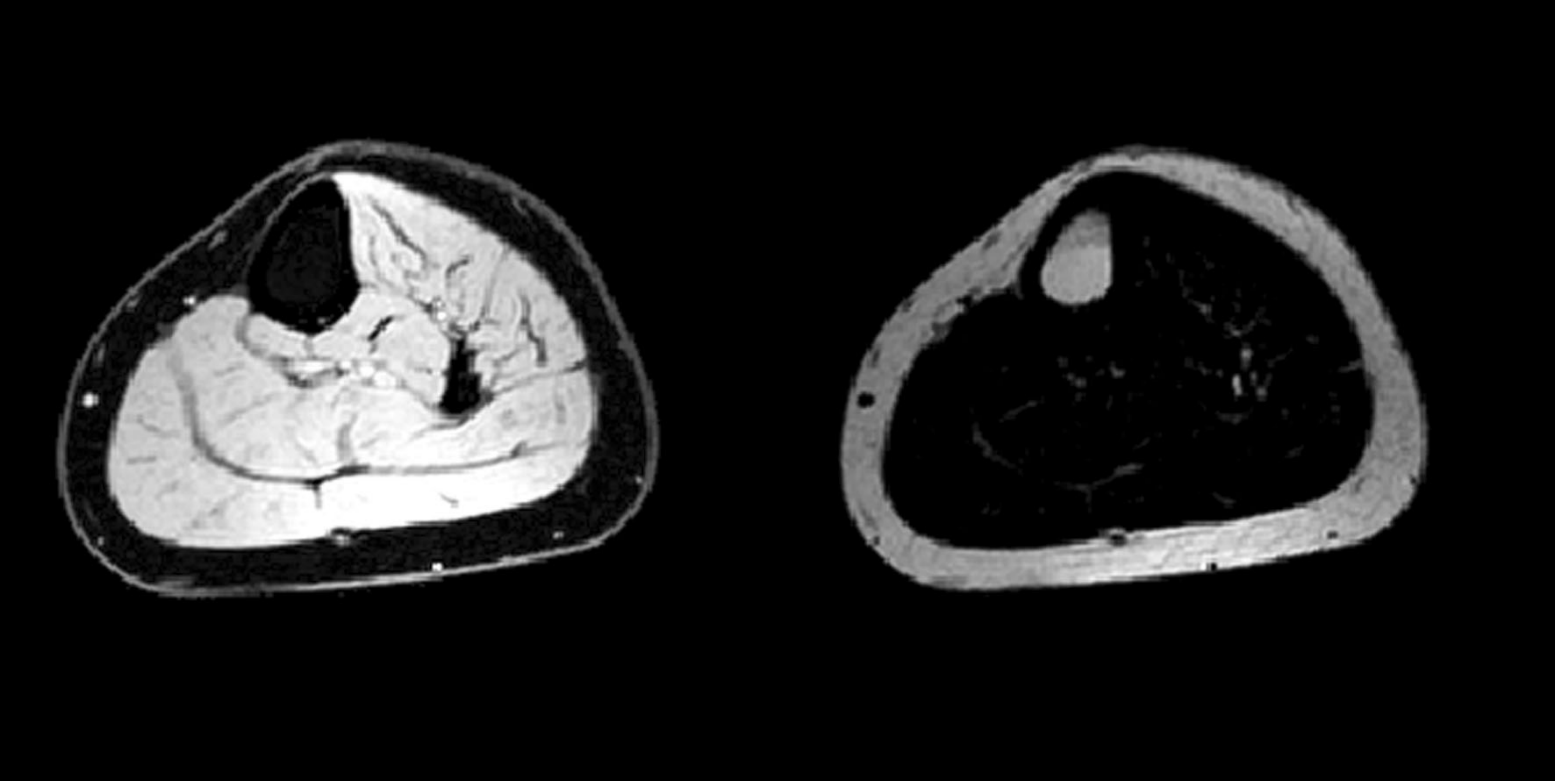
Use of a mathematical model enables the signal from the water components and the fat components to be separated, though these images still contain B_1_ inhomogeneity

**Fig. 4:**
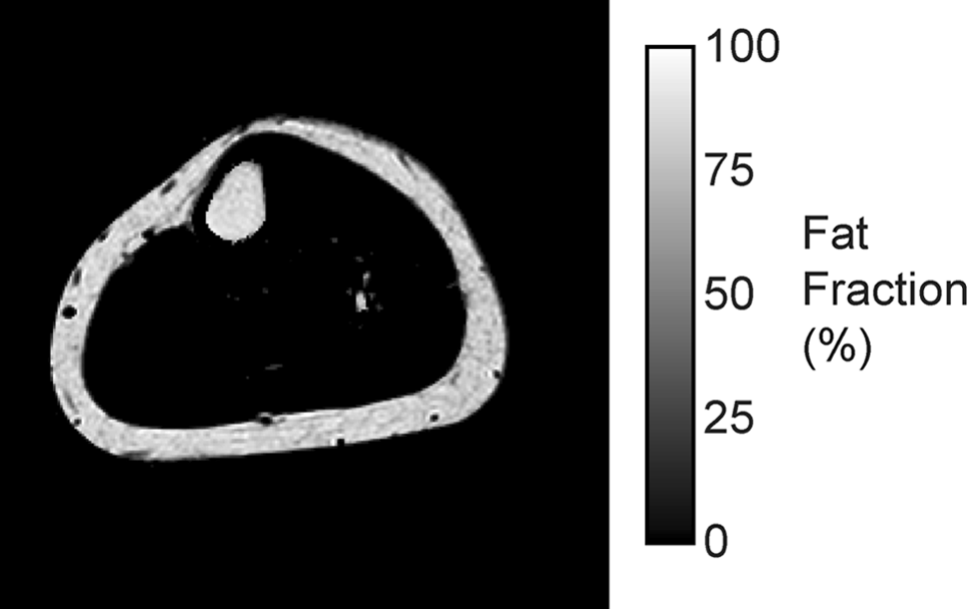
If we calculate the percentage of fat signal in the total MR signal, then the background inhomogeneity disappears and we are left with a map of fat fraction from 0 to 100% which is comparable between scan sessions and individuals

The protons within water and lipids resonate at slightly different frequencies. For example, the CH_2_ groups in the fatty acid chains resonate at a lower frequency than water. In a gradient echo scan, this means that though the CH_2_ and water spins start off in phase immediately after the initial excitation pulse, like two men running on a circular racetrack at different speeds, there is an echo time when the signals from water and CH_2_ are on the opposite side of the racetrack, that is to say 180° out of phase and their signals will cancel. By acquiring an image after twice this echo time has elapsed, the water and CH_2_ would be back in phase, and their signals will add (Fig. 2). In principle, by acquiring images at the two time points described and by adding and subtracting the signal, we can produce images purely of fat and of water (Fig. 3). The respective fat and water signals can then be used to calculate the fat fraction, expressed as the fraction of fat signal in the total signal in each voxel (Fig. 4):

$$ \eta = \frac{{S_{\text{F}} }}{{S_{\text{F}} + S_{\text{W}} }} $$

where *η* is the fat fraction, and *S*_F_ and *S*_W_ are, respectively, fat and water signals [15].

One major limitation of this simple, so-called, two point Dixon approach [16] is that it assumes that the magnetic field is homogeneous everywhere, which is generally not true. However, this complication can be accommodated by acquiring a third echo [17], giving rise to the so-called three-point Dixon method. Although the original papers describing this approach envisaged using purely in phase and out of phase echo times, this is not mandatory and the fat and water signals can be separated using other echo times with a more general signal model [18].

Most work in neuromuscular diseases has been performed with either 2 or 3 gradient echoes, sometimes collected within a single TR (which is faster) or sometimes in separate TRs. More than three echoes can be collected for better signal-to-noise ratio and for improved precision of the fat fraction measurement (see “Advanced: choosing echo times and signal efficiency”).

### What to look out for when processing quantitative fat imaging

While calculating the fat fraction from the water and fat images may seem straightforward, there are several confounding factors to consider. If the measure is going to be used as a biomarker, particularly in multi-centre studies, it should be accurate, reproducible, and independent of the precise scan parameters used. For this reason, the proton density fat fraction (PDFF) (which has been previously recommended for liver studies) is a suitable measure. PDFF is the ratio of the density of mobile triglyceride protons to the total density of mobile protons that are MR visible [15, 19, 20]. Note that the PDFF is not an absolute concentration of fat, but a relative one. In the presence of edema and fibrosis, the measured water signal is not the same as the absolute water content of the voxel. While it can be shown that the PDFF is linearly correlated to the mass fat fraction, the correlation coefficient strongly depends on the tissue studied; PDFF can serve, however, as an independent biomarker in its own right [19].

There are several major confounding factors that need to be accounted for and mitigated: Table 1 lists the main factors, their effects, and suggested solutions. In summary, a properly implemented fat–water separation method with sufficient long TR and low FA to minimise T1-weighting, three or more echoes, using multi-spectral modelling of fat and T2* correction can yield a quantitative value that can be compared both longitudinally and cross-sectionally between centres subject to appropriate quality assurance procedures (“Quality assurance”).

**Table 1:** Confounders to fat fraction analysis

Confounding factor	Origin	Effect if ignored	Solution
Acquisition sequence is T1-weighted	Water and fat have very different T1 relaxation times in muscle	The reported fat fraction is artificially high	(a) Avoid T1-weighting using long TR and low flip angle [64] (b) Post-processing correction with pre-calibrated data [15, 65]
Fat spectrum is not fully represented by CH_2_ alone ~30% of the proton signal lies away from the CH_2_ peak – Fig. 5 [12, 62, 66]	The lipid resonance has multiple resonant frequencies other than the CH_2_ peak (Fig. 5)	The reported fat fraction is artificially low	Use a fat–water separation model that permits a multi-spectral model, containing typically 6 or 9 spectral components [67, 68]
Not accounting for T2* relaxation	The effective T2^*^ produces weighting in a series of gradient echo images with varying TE. T2^*^ varies with muscle involvement	Low fat fractions have positive bias, uncertainty in PDFF at low fat fraction [43]	Use a fat–water separation model that allows a single T2^*^ component to be specified [43]
Biased fat fractions near PDFF ≈ 0% and 100%	Noise bias caused by magnitude correction	Fat fractions positively or negatively biased	Calculate the PDFF using a noise bias correction method [64]
Phase inaccuracy	Bipolar readouts, eddy currents, diffusion gradients [15, 20, 64, 69, 70]	Artefacts in the fat fraction map	(a) Use sequences with monopolar readouts (b) Correct modelling for eddy currents or bipolar readouts

### Advanced: choosing echo times and signal efficiency

The echo spacing for the traditional gradient echo in and out of phase images can be worked out as follows: with a 435 Hz difference at 3.0 T, the signals are in phase 435 times per second, so after the start, they are in phase again after 1/435 s = 2.3 ms; thus, they will be out of phase for the first time at 1.15 ms and every 2.3 ms after that. The signal-to-noise efficiency of Dixon imaging can be increased by varying the echo spacing, so that the intervals no longer correspond to the in and out of phase echo times. Reeder and colleagues introduced this concept by calculating the echo intervals that maximise the effective signal to noise across the range of fat fractions for a multi-echo acquisition [18, 21]. When combined with an iterative algorithm for the fat–water separation, this approach is known by the acronym IDEAL (iterative decomposition of water and fat with echo asymmetry and least-squares estimation). However, the purpose remains the same as the 2 and 3 point Dixon techniques.

### Measuring T2 and PDFF with the same sequence

The majority of 2 and 3-point Dixon applications in skeletal muscle have used gradient echo readouts. However, spin-echo (SE) and fast spin-echo (FSE) may also be used as originally proposed by Glover and Schneider [17]. One possible factor contributing to limited use of spin-echo Dixon techniques is increased scan times due to prolongation of the repetition time for multi-slice imaging [22].

Often, PDFF and the T2 relaxation time of water, a surrogate for inflammation and edema, are measured using two different sequences. The efficiency of the scan can be improved by measuring them using the same sequence. IDEAL fat–water separation has also been combined with a CPMG spin-echo train in the so-called IDEAL-CPMG sequence [23]. Although IDEAL-CPMG is thus far not widely available, this approach enables independent measurements of the T2-relaxation times of the separated fat and water components in skeletal muscle, an important capability in conditions where water- and fat-based pathologies may exist simultaneously [24, 25].

## T2-corrected spectroscopy to measure muscle fat content

**Fig. 5:**
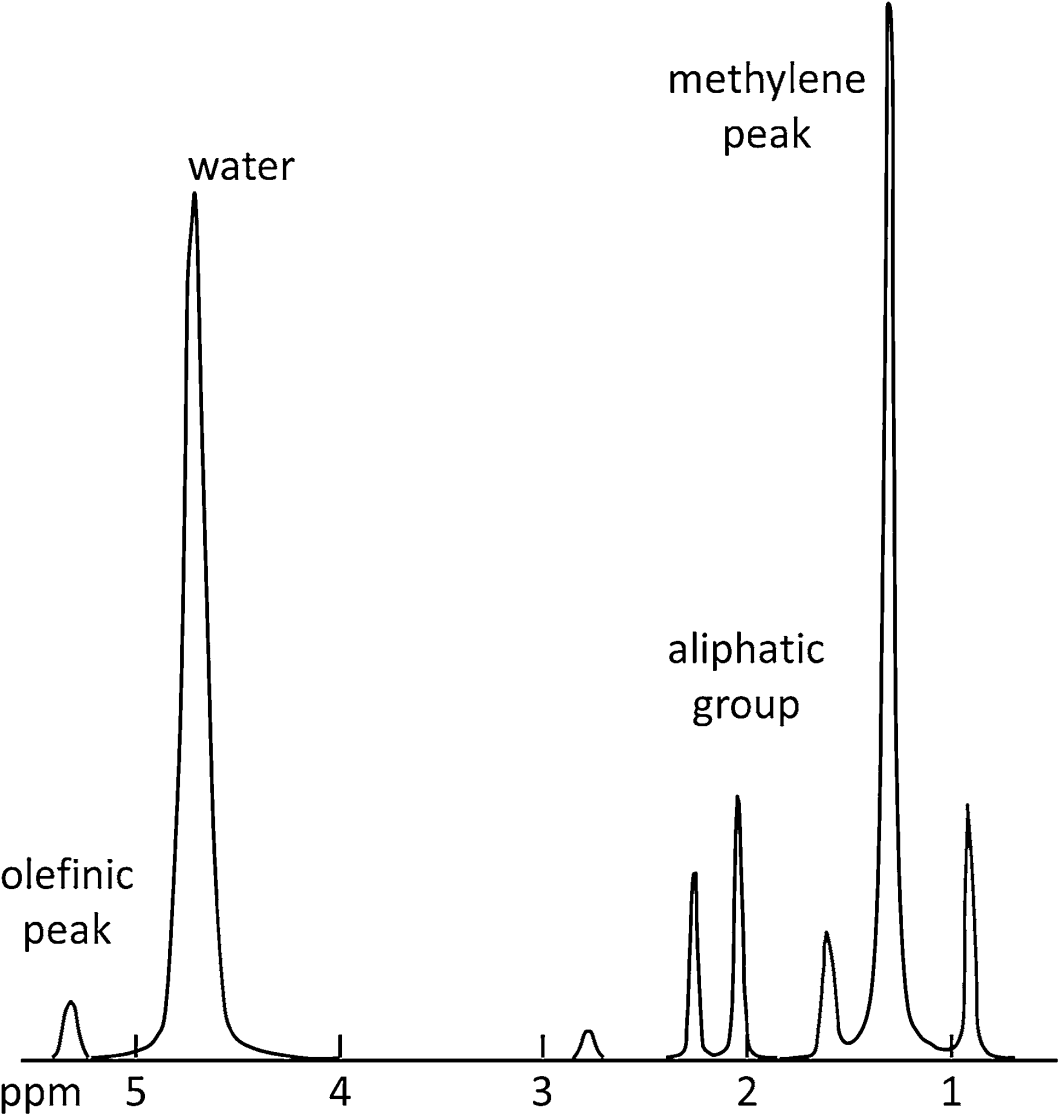
Typical water–fat spectrum based on ref 68. Fat signal is often modelled on one off-resonant frequency (1.3 ppm); however, this is not accurate, since up to 30% of fat signal may lie at different locations. For true quantitative measurements, the entire fat spectrum should be accounted for

Traditionally, spectroscopic methods have been used to determine changes in lipid content (both intramyocellular (IMCL) and extramyocellular (EMCL) lipids), lipid saturation, and major muscle metabolites including trimethyl ammonium compounds (e.g., choline and carnitine), creatine, carnosine, acylcarnitines, and lactate [26]. Proton MRS can also be used to determine absolute tissue water and lipid content with high accuracy and reproducibility using both internal and external referencing methods [27, 28]. In the presence of fibrosis and/or edema, commonly found in muscular dystrophies, absolute water content can only be obtained using an external reference method. However, this is currently not commonly applied. As described above for the imaging methods, corrections for T2 and T1 relaxation need to be applied for the individual metabolites to calculate a PDFF. In addition, the relative contribution of each resonance to the overall lipid content signal (Fig. 5) needs to be determined. MRS determined fat fractions have become the standard against which to validate PDFF calculations based on imaging techniques [29–31]. An advantage of MRS is that the fat fraction measurement is made directly from the spectral peaks representing the lipids present. In imaging methods, a fixed lipid spectral model of lipid is necessary. Therefore, information can sometimes be obtained about triglyceride properties with MRS, including the level of carbon chain unsaturation. Whereas high-resolution MR spectroscopy will resolve all the major lipid resonances, at clinical field strengths, individual lipid resonances may need to be deconvolved using a number of different spectral processing techniques [32].

Whereas accelerated multi-voxel spectroscopic imaging techniques have been developed [33] and implemented in muscle [34], the spatial resolution is less than that obtained with MRI and 3D coverage has been limited by long acquisition times. Signal bleeding from neighboring voxels can influence accuracy. Hence, most MRS measures of PDFF have been based on targeted, single voxel spectroscopy [10, 35–38]. A disadvantage of this localization method is the relatively large voxel size; voxels are typically an order of magnitude bigger in size than MRI, and range from 5 to 20 ml. While this is beneficial from a signal-to-noise ratio (SNR) perspective and enhances greater detection sensitivity to low fat concentrations, it requires accurate voxel placement and extensive operator training. In practice, it is feasible to obtain MR spectra of only a couple of selected muscles within a single examination.

## Studies which have used quantitative fat fraction measurements in muscular dystrophy

The first study using the Dixon approach to examine the fat content of dystrophic muscle in humans was published in 2008 (Table 2). In the period since then, and particularly in the last 4 years, qMRI methods to quantify fat replacement have been performed in many neuromuscular diseases. It is arguable that this work has been stimulated by the prospect of first-in-human trials in some conditions, particularly DMD. In this section and the adjoining tables, we summarise the studies that have been undertaken.

**Table 2:** Cross-sectional cohort studies

Study	Field (T)	Population	No. of patients	Method	Multi-spectral model?	T2* corrected?	Correlations?
Fischmann et al. [5]	1.5	OPMD	8	2 point	No	No	Function
Fischmann et al. [71]	3.0	DMD	20	2 point	No	No	Function
Forbes et al. [37]	3.0	DMD	123	MRS	n/a	n/a	N/a
Gaeta et al. [72]	1.5	DMD	20	2 point	No	No	Function
Hooijmans et al. [46]	3.0	DMD	18	3 point	Yes	No	T2,^31^P MRS
Hooijmans et al. 2017 [73]	3.0	DMD	22	3 point	Yes	No	Modelling of non-uniformity of fat replacement in proximodistal axis
Horvath et al. [74]	3.0	LOPD	7	2 point	Yes	Yes	Function
Lokken et al. [41]	3.0	BMD	14	2 point	n/k	n/k	Muscle strength to cross-sectional area
		LGMD2I	11				
Mankodi et al. [24]	3.0	DMD	13	3 point IDEAL-CPMG	Yes	n/a	Use of IDEAL-CPMG sequence to measure fat fraction and T2 in Duchenne. Small longitudinal follow-up group
van den Bergen et al. 2014 [75]	3.0	BMD	9	3 point	Yes	No, global T2 correction	Dystrophin levels
Willcocks et al. [52]	3.0	DMD	22	MRS and 3 point	Yes	Yes	Performance of upper limb test, grip strength
Willis et al. [40]	3.0	LGMD2I	38	3 point	No	No	Function
Wokke et al. [76]	3.0	BMD	25	3 point	Yes	No, global T2 correction	Function and^31^P MRS
Wokke et al. [39]	3.0	DMD	16	3 point	Yes	No, global T2 correction	Function
Wren et al. [77]	1.5	DMD	9	3 point	No	No	Function

*n/a* not applicable, *n/k* not known from manuscript, *OPMD* oculopharyngeal muscular dystrophy, *DMD* Duchenne muscular dystrophy, *LGMD2I* limb girdle muscular dystrophy 2I, *BMD* Becker muscular dystrophy, *LOPD* late-onset Pompe’s disease (glycogen storage disease type II)

In the tables, the studies have been categorised in three groups. First, those studies which have been undertaken on a cross-sectional cohort of a particular form of muscular dystrophy are shown in Table 2. These studies have typically been performed to determine the pattern of fat replacement within a cohort and to describe cross-sectional changes with increasing age. The main focus of these studies has been on the dystrophinopathies. In general, the involvement pattern varies considerably between different diseases, where one muscle can be affected early in one disease and be spared until late in the disease process in another. A typical example is the clear involvement of the soleus muscle in the calf of OPMD patients [5], while this muscle is involved only after the tibialis anterior and peroneal muscles become affected in DMD [39]. In addition to differences between diseases, within a particular disease with the same underlying genetic defect, the fatty replacement of individual muscle groups can vary widely, as demonstrated in a multi-national LGMD2I cross-sectional study [40]. Often, the fat fraction measurements from individual muscle groups have been correlated against physical function tests. In general, fat fractions correlate well with functional tests. However, especially in DMD and BMD, it has been shown that contractile properties are disrupted, because specific force is reduced [39, 41]. This type of analysis is performed using Dixon imaging to generate a maximal contractile cross-sectional area and then comparing this to muscle strength measurements.

**Fig. 6:**
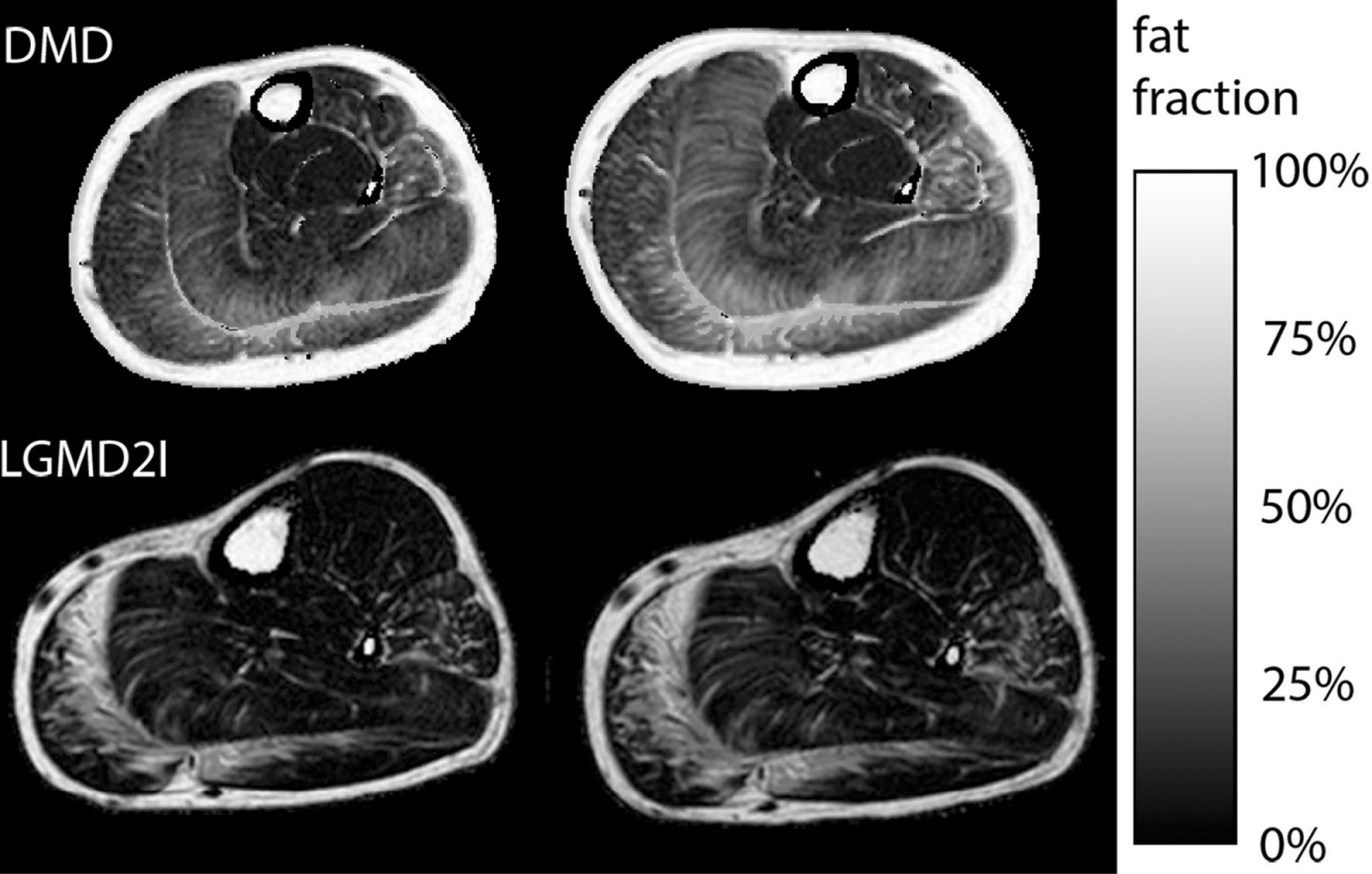
Example of using PDFF maps to assess progression at baseline (*left*) and 12 months later (*right*) in the lower leg of a patient with limb girdle muscular dystrophy 2I (*bottom*) and Duchenne muscular dystrophy (*top*). Progression in 1 year is generally much more rapid in DMD compared to LGMD2I: fat fraction changes measured included the soleus (21–28% in DMD, 8–13% in LGMD2I), tibialis anterior (12–16% in DMD, no change at 6% in LGMD2I), lateral gastrocnemius (21–29% in DMD, 20–24% in LGMD2I), medial gastrocnemius (15–20% in DMD, 29–49% in LGMD2I), and the peroneus (28–36% in DMD, 18–26% in LGMD2I)

To prepare qMRI end-points for clinical trials, it is important to know in which muscle groups fatty replacement will progress in the absence of remedial treatment, as rates of progression vary between different muscle groups and over time. The most suitable muscle group to follow will depend on several factors, including age, type of disease, and mechanism of action of the drug. For instance, the efficacy of a drug that is thought to prevent a further increase in fat replacement would be most suitable to test in a muscle and age group where there is a clear increase in fat replacement over time. This muscle group will then have the highest standardized response mean. In DMD, for instance, it has been suggested that between the ages of 9 and 11 years, the vastus lateralis has the highest standardized response mean [10]. On the other hand, a drug that aims to increase muscle mass can also be evaluated in a muscle that does not show fat replacement yet, merely by assessing at the contractile volume. Table 3 outlines the longitudinal studies that have been performed in different muscular dystrophies to measure the rates of progression. Figure 6 shows two examples of fat fraction progression across 1 year in a slow-progressing muscular dystrophy, LGMD2I [11], and one in the more rapidly progressing DMD. Most of these studies have examined the natural history of different diseases, though Arpan et al. 2014 considered the effects of corticosteroid initiation in DMD boys [35] and Carlier et al. 2015 [42] examined the effect of enzyme replacement therapy in late-onset Pompe’s disease. Commonly, these longitudinal studies contrast the increase in muscle fat fraction revealed by qMRI and disease progression demonstrated by the standard physical function tests that are routinely used in clinical care and clinical trials. These studies have demonstrated the enhanced sensitivity of qMRI compared to the existing measurements in both the legs [9–11, 24, 43] and the arms [8, 44].

**Table 3:** Studies with longitudinal data measuring fat fraction

Study	Field (T)	Population	No of patients	Method	Multi-spectral model?	T2^*^ corrected?	Longitudinal interval(s)	Correlations
Andersen et al. [78]	3.0	FSHD	45	2 point	n/k	n/k	1 year	Function
Arpan et al. [35]	3.0	DMD	15	MRS	n/a	n/a	3 months, 6 months, 1 year	Corticosteroid use, function
Bonati et al. [79]	3.0	DMD	20	2 point	No	No	1 year	Motor function
Bonati et al. [80]	3.0	BMD	3	2 point	No	No	1 year	
Bonati et al. [81]	3.0	SMA	18	2 point and 6 point	No Yes	No Yes	3 months, 6 months, 1 year	Function, molecular biomarkers
Carlier et al. [42]	3.0	LOPD	23	3 point	n/k	n/k	1 year	Enzyme replacement therapy
Fischmann et al. [7]	1.5	OPMD	5	2 point	No	No	13 months	Function
Hogrel et al. [8]^a^	3.0	DMD	25	3 point	n/k	n/k	1 year	Function
Morrow et al. [9]	3.0	CMT1A IBM	20 20	3 point	n/k	n/k	1 year	Function
Ricotti et al. [44]^a^	3.0	DMD	15	3 point	No	No	3 months, 6 months, 1 year	Performance of upper limb, pinch strength
Wary et al. [54]^a^	3.0	DMD	24 (9)	3 point	n/k	n/k	1 year	Ambulation
Willcocks et al. [10]	3.0	DMD	109	MRS	n/a	n/a	3 months (*n* = 11) 6 months (*n* = 15) 1 year	Function
Willis et al. [11]	3.0	LGMD2I	32	3 point	No	No	1 year	Function, FVC

*n/a* not applicable, *n/k* not known from manuscript, *FSHD* facioscapulohumeral muscular dystrophy, *OPMD* oculopharyngeal muscular dystrophy, *DMD* Duchenne muscular dystrophy, *LGMD2I* limb girdle muscular dystrophy 2I, *BMD* Becker muscular dystrophy, *LOPD* late-onset Pompe’s disease (glycogen storage disease type II), *CMT1A* Charcot-Marie-Tooth disease 1A, *IBM* inclusion body myositis, *SMA* spinal muscular atrophy

^a^Study of the upper limb

Finally, some studies on neuromuscular disease have been performed to investigate new MR methods in these patients, but contain valuable information about patient cohorts (Table 4). These include quality assurance and reproducibility studies [25, 38], work to accelerate image acquisition [43, 45], and correlations between MRI and MRS methods [38, 46].

**Table 4:** Studies principally concerning MR methodology which contain patient populations

Study	Field (T)	Population	No. of patients	Method	Multi-spectral model?	T2* corrected?	Study topic
Azzabou et al. [82]	3.0	Multiple	48	3 point	no, post hoc adjustment	no	Validation of a three exponential model for fitting multi-echo T2 data
Forbes et al. [36]	3.0	DMD	30	MRS	n/a	n/a	Reproducibility study
Gloor et al. [13]	1.5	OPMD	8	2 point	No	No	Comparison of fat imaging techniques
Hollingsworth et al. [45]	3.0	BMD	8	3 point	Yes	No	Scan acceleration by compressed sensing techniques
Hooijmans et al. [83]	3.0	DMD	24	3 point	No	No	Diffusion tensor imaging
Lareau-Trudel et al. [56]	1.5	FSHD	35	T1-weighted	No	No	Automated evaluation
Loughran et al. [43]	3.0	BMD	8	3 point, 6 point	Yes	Yes	Scan acceleration by compressed sensing techniques, role of T2* correction
Mankodi et al. [24]	3.0	DMD	13	3 point IDEAL-CPMG	Yes	n/a	Use of IDEAL-CPMG sequence to measure fat fraction and T2 in Duchenne. Small longitudinal follow-up group
Marty et al. [84]	3.0	Mixed	22	3 point	No, post hoc adjustment	No	Validation of extended phase graph method for fitting multi-echo T2 data
Ponrartana et al. [85]	3.0	DMD	13	6 point	Yes	Yes	Diffusion tensor imaging
Sinclair et al. [25]	3.0	HypoPP	12	3 point IDEAL CPMG	Yes	n/a	Stability and sensitivity of IDEAL-CPMG sequence to measure fat fraction and T_2_
Triplett et al. [38]	3.0	DMD^a^,	71^a^	3 point, MRS	Yes	No	Correlation of MRI and MRS methods
		COL6^b^	16^b^				
Wokke et al. [12]	3.0	DMD	13	3 point	Yes	No, global T2 correction	Comparison of multi-spectral models and qualitative grading systems

*n/a* not applicable, *n/k* not known from manuscript, *OPMD* oculopharyngeal muscular dystrophy, *DMD* Duchenne muscular dystrophy, *BMD* Becker muscular dystrophy, *LOPD* late-onset Pompe’s disease (glycogen storage disease type II), *COL6* collagen VI deficiency, *HypoPP* hypokalemic periodic paralysis, *FSHD* facioscapulohumeral muscular dystrophy

## Quality assurance

PDFF as determined by MRI is a derived value based on multiple analytical steps (see “Quantitative fat fraction imaging measurements: how is it done?”). In the pure research environment, each of these steps may be continually monitored and quality controlled using validation data test sets and tissue phantoms. This might not always be possible in the purely clinical environment in which fat/water maps are provided solely as derived DICOM images generated by vendor specific software. As outlined in “What to look out for when processing quantitative fat imaging” and Table 1, techniques for separating fat and water have evolved to include more complex models for lipids as well as to take into T2* relaxation into account. Therefore, it is essential to continually monitor these derived values using known standards to address both the accuracy and precision of the PDFF measurements. Whereas monitoring the precision of the PDFF within a single site may be sufficient, clearly a high level of accuracy is essential for continuity between sites and studies in longitudinal, multi-site trials. During a quality control and validation stage, accuracy and precision can be readily tracked and documented using a combination of MRS and MRI methods on both phantoms and human test subjects. Some clinical trials that use MRI as an end-point now demand that a calibrated test object is scanned before or after every subject visit to monitor system and measurement stability.

Whereas there is currently no standard for quality control measures for PDFF MRI determination, a number of viable options exist.

### Intralipid-based phantoms

Similar to common T1 and T2 calibration phantoms, vials containing different PDFF values can be made. A simple phantom can be constructed from a copper sulphate solution, pure soybean oil, and commercially available Intralipid (Registered-Baxter Healthcare Corporation) emulsions at different concentrations (available at 10 and 20%). Analysis of the images will yield a reference curve that can be matched to single MRS acquisitions to determine the linearity and agreement between MRS and MRI PDFF. This tissue phantom is easy to create and allows for testing changes in T1 and T2* in the aqueous solutions.

### Fat/water container

For example, a sample bottle in which two layers of fluid have been introduced, water and oil, to form two immiscible layers, with no emulsified droplets at the boundary. For 2D acquisitions, images can be acquired, such that the slice thickness progressively moves through the water/fat interface, by an increment of 0.5–1 mm each time [47]. This will build up a series of images where the composition of the slice changes from 0% fat to 100% in known increments. Another option is to use an angled slice through the fat/water interface; in this way, a gradient of PDFF can be created from 0 to 100% [38]. Analysis of these images can yield a reference curve that can be directly matched to single voxel MRS acquisitions to determine the linearity and agreement between MRS and MRI determined PDFF. This phantom does not allow testing of reconstruction algorithms that take into account differences in T1 and T2* on the derived PDFFs.

### Customized water–fat emulsions and agar gels

Water and fat emulsions for PDFF calibration have been created from soya and carrageenan [48] or peanut oil and 2% agar [49]. To achieve homogenous mixing and stability, it is necessary to add surfactant and sodium azide to the emulsion. This phantom has the advantage that different concentrations of superparamagnetic iron oxide can be mixed in to provide T2* weighting at the different fat fractions ranging from 0 to 100%. Moreover, this phantom has the advantage that variable amounts of heavy water (D_2_O) can be used as the solvent to simulate decreased H_2_O proton density observed on the calculated PDFF [50]. Similar to above, accuracy and precision can be determined by determining the relationship between MRS and MRI derived PDFFs.

## Patient placement, acquisition time and scan acceleration, practical issues and segmentation

For leg imaging, the preferred positioning is feet-first supine, with the legs stretched, the patella facing upward, and the ankles in the neutral position (i.e., minimal exo or endorotation). To enhance comfort, a small pillow can be placed under the knees, but the standard leg rest delivered with many scanners will not accommodate this, and the sides of the ankles can be supported with sandbags to prevent unintended movements. In patients with contractures positioning with stretched legs and neutral ankles is not always feasible, and care should be taken to provide optimal support for comfort. Positioning of the upper limb is more challenging, as scan quality is severely reduced at the side of the magnet bore. Experience with scanning of this body part is limited [8, 44, 51, 52], but it will become more common with the need to develop outcome measures for non-ambulant patients. The so-called ‘superman’ position [53] is likely impossible for patients with MDs, and hence, optimal positioning will either be (a) in the fetal position, (b) supine with the upper limb along the trunk, while the trunk is as far off-centre as possible [8, 44, 52, 54], or (c) supine with a dedicated arm rest placed over the abdomen of the patient: all published studies in DMD have so far selected the second option.

Imaging can be performed in 2D or 3D, with slices in the transverse plane perpendicular to the bone to facilitate analysis. The location of the slice stack should be recorded precisely to enable longitudinal measurements, for instance at a fixed distance from a bony landmark, such as the tibial plateau or the patella. Landmarking can either be done during the imaging session using a scout image [5] or prior to the scanning session by placing a fish oil capsule on the skin at a pre-defined distance from surface anatomy landmarks like the iliac crest [55]. Receiver coils can either be array coils for wide coverage of the lower limb or local surface coils. The latter may be more convenient for imaging the upper limb and for patients with contractures.

As fat replacement of muscle tissue can be non-uniform along the muscle length [51], it is important that the slice stack covers as much of the muscle as possible. Commonly, this is achieved by selecting a field of view from joint to joint, i.e., malleolus to patella for the leg, and patella to iliac crest for the thigh. Slice thickness is usually between 2 and 10 mm with a 0–5 mm gap, resulting in 10–50 slices per body part. This poses a significant burden in terms of data processing as traditionally, data are being processed by outlining every single muscle in a pre-defined number of slices by a trained operator—which is a labour intensive process. In the last decade, several techniques have been developed that can quantify total adipose tissue volume and total muscle volume based on either T1 weighted or Dixon images [52, 56–58]. In these approaches, the total muscle volume of an entire slice stack can automatically be segmented, thereby severely reducing the processing time. Unfortunately, severe fatty replacement is still a problem, as it is difficult to separate fat replacement from subcutaneous fat, and hence, user input is required to solve this [56, 59]. More importantly, as fatty replacement occurs in a muscle specific way, it is preferable to obtain information of each muscle separately instead of the entire muscle volume—and this is not possible using this methodology. While, semi-automatic approaches have been developed that can segment individual muscles [60, 61], these have not been applied in NMDs yet. In the absence of such an approach, segmentation is unfortunately still often performed manually and the field is in dire need of adequate approaches to solve this.

Depending on the choice of repetition time and number of signal averages used, a common Dixon acquisition of the entire upper leg can take 3–5 min. Using scan acceleration techniques, acquisition time can be reduced dramatically to about 1 min [43, 45]. However, these techniques are not yet standard on clinical systems.

## Vendor implementations of quantitative protocols to measure PDFF

The basic types of multi-echo acquisition sequences described in “Quantitative fat fraction imaging measurements: how is it done?” can be achieved by most clinical MRI scanners in skeletal muscle, even where the echoes have to be collected in separate acquisitions. Historically, it has not been possible to buy directly from the vendor an integrated system that will both perform the data acquisition and reconstruct the proton density fat fraction images at the time of acquisition. Most of the studies described in “Studies which have used quantitative fat fraction measurements in muscular dystrophy” have extracted the complex image data from the scanner and applied custom algorithms, such as those described in [15, 17, 62] to separate the fat and water and produce fat fraction images. Of particular note was the ISMRM Workshop on Water–fat separation, which collated examples of MATLAB code which can achieve this task and have been made available [15].

Of course, the methods for measuring fat fraction have also found extensive use in the measurement of hepatic steatosis in type 2 diabetes and non-alcoholic fatty liver disease [63]. Given the size of these markets, the main MRI vendors have now released products, usually sold as optional upgrades to the base scanner hardware, which claim to reliably measure hepatic steatosis: IDEAL-IQ (GE), mDixon Quant (Philips), and LiverLab (Siemens). However, it is important to note that these products and their optimised settings have not been validated in skeletal muscle and it should not be assumed that they will automatically give acceptable answers outside of their intended terms of use. The same quality assurance procedures outlined in “Quality assurance” should be adopted and consideration should also be given to the effect of a change of vendor software during the course of a longitudinal study.

## Summary

Characterising rare muscle diseases with qMRI is an important task, because these techniques may be substantially more responsive than the existing clinical measures of function currently used as end-points in clinical trials [9–11]. However, there are various qMRI approaches available that are influenced by details of acquisition and reconstruction, and this should be taken into consideration when comparing results from different centres. In this paper, we have given a conceptual and practical introduction to Dixon fat fraction imaging and MRS, drawing attention to the advantages and potential confounding factors of each. The authors believe that fat fraction imaging and MRS will both have an important role to play in future scientific and therapeutic studies, with quantitative MRI methods more familiar to operators in most institutions. Regardless of the method used, adequate operator training, qualification, and quality assurance processes will be needed over and above those used for standard radiological imaging. This will demand specialist input to co-ordinate and oversee clinical trials involving quantitative MRI. Given the low prevalence of many of the muscular dystrophies, high quality data may demand that patients travel to a smaller number of centres specialising in quantitative MRI. Many new clinical trials for DMD now require quantitative MRI in at least a subset of patients.

Practical considerations for quality assurance, patient positioning, slice prescription, and the available vendor systems were introduced. By summarising the available literature describing the application of Dixon-based fat fraction imaging in muscle dystrophies, we hope to highlight the power and promise of these techniques, while emphasising that a pragmatic consideration of the limitations is essential to maximise the potential advantages that fat fraction imaging confers on longitudinal clinical trials in this important class of diseases.

The authors would suggest that, with the increasing availability of quantitative MRI methods on modern scanners, and the increasing bodies of longitudinal literature identifying target muscle groups to study in therapeutic interventions, the quantitative MRI methods are reaching maturity and will have a critical and central role to play and end-points in future therapeutic trials.
